# Carcass Persistence and Detectability: Reducing the Uncertainty Surrounding Wildlife-Vehicle Collision Surveys

**DOI:** 10.1371/journal.pone.0165608

**Published:** 2016-11-02

**Authors:** Rodrigo Augusto Lima Santos, Sara M. Santos, Margarida Santos-Reis, Almir Picanço de Figueiredo, Alex Bager, Ludmilla M. S. Aguiar, Fernando Ascensão

**Affiliations:** 1 Department of Zoology, University of Brasília-UnB, Brasília, Federal District, Brazil; 2 Centre for Ecology, Evolution and Environmental Changes, Faculty of Sciences, University of Lisbon, Lisbon, Portugal; 3 IBRAM—Instituto Brasília Ambiental, Brasília, Federal District, Brazil; 4 CIBIO-UE–Research Centre in Biodiversity and Genetic Resources, Pole of Évora, Research Group in Applied Ecology, University of Évora, Évora, Portugal; 5 UBC–Conservation Biology Lab, Department of Biology, University of Évora, Évora, Portugal; 6 Department of Biology, Federal University of Lavras, Lavras, Minas Gerais, Brazil; 7 Infraestruturas de Portugal BiodiversityChair. CIBIO/InBio, Centro de Investigação em Biodiversidade e Recursos Genéticos, Universidade do Porto. Campus Agrário de Vairão, Vairão, Portugal; 8 CEABN/InBio, Centro de Ecologia Aplicada “Professor Baeta Neves”, Instituto Superior de Agronomia, Universidade de Lisboa, Tapada da Ajuda, 1349–017 Lisboa, Portugal; INIBIOMA (Universidad Nacional del Comahue-CONICET), ARGENTINA

## Abstract

Carcass persistence time and detectability are two main sources of uncertainty on roadkill surveys. In this study, we evaluate the influence of these uncertainties on roadkill surveys and estimates. To estimate carcass persistence time, three observers (including the driver) surveyed 114km by car on a monthly basis for two years, searching for wildlife-vehicle collisions (WVC). Each survey consisted of five consecutive days. To estimate carcass detectability, we randomly selected stretches of 500m to be also surveyed on foot by two other observers (total 292 walked stretches, 146 km walked). We expected that body size of the carcass, road type, presence of scavengers and weather conditions to be the main drivers influencing the carcass persistence times, but their relative importance was unknown. We also expected detectability to be highly dependent on body size. Overall, we recorded low median persistence times (one day) and low detectability (<10%) for all vertebrates. The results indicate that body size and landscape cover (as a surrogate of scavengers’ presence) are the major drivers of carcass persistence. Detectability was lower for animals with body mass less than 100g when compared to carcass with higher body mass. We estimated that our recorded mortality rates underestimated actual values of mortality by 2–10 fold. Although persistence times were similar to previous studies, the detectability rates here described are very different from previous studies. The results suggest that detectability is the main source of bias across WVC studies. Therefore, more than persistence times, studies should carefully account for differing detectability when comparing WVC studies.

## Introduction

Roads and associated traffic promote a variety of negative effects on biodiversity, including habitat degradation and pollution, dispersal of exotic species, and barrier effects [1–5]. Wildlife-vehicle collisions (WVC), however, are often recognized as the most important source of non-natural animal mortality, exceeding other significant impacts such as hunting [2, 6, 7]. Population declines, inbreeding depression and local extinctions of some species may occur due to roadkills [1, 4, 8, 9]. In fact, virtually all species using road vicinities are negatively affected by WVC, from insects [10] to all terrestrial vertebrates [11–15]. To avoid these negative effects, mitigation measures such as faunal passages and drift fencing [2,4,5,6] are generally applied at road sections with higher frequencies of roadkills[14]. Because these mitigation measures are often expensive, it is crucial that roadkill rates along the road network are properly quantified for a correct identification of most problematic road sections [16–18].Besides, correcting mortality estimates is very important to assess the effects of roadkills on population depletion. This, requires accurate WVC estimates, correcting for the two main sources of bias: carcass persistence time and carcass detectability [16–18]. Yet, the use of such unbiased estimates has barely been used[16, 18, 19].

Persistence time is the period up to which a carcass remains detectable, i.e. before it is decomposed by traffic or removed by scavengers [20], and is influenced by several factors, including the size of the carcass, traffic volume, and weather conditions [18, 21–27]. Larger carcasses are expected to remain for longer periods, while roads with higher traffic volume are expected to reduce carcass persistence given the faster degradation of more vehicles passing by [18,23,26]. Regarding weather, during the rainy season it is expected that carcasses show shorter persistence times, since heavy rain also promotes faster degradation of carcass, and washes away carcass debris [23, 26]. On the other hand, in drier days and at higher temperatures carcass may suffer desiccation therefore increasing the persistence time [23, 26]. Another important source of variation in persistence time is the scavenging activity, which is naturally related to the abundance and diversity of scavengers inhabiting the roads’ vicinity areas [1,18,26].The main difficulty in assessing the importance of scavenging for carcass persistence is obtaining reliable estimates of abundance and activity of scavengers in the vicinity of roads. One option to circumvent this difficulty is to use proxies for scavengers presence. The abundance and diversity of scavengers is expected to be higher in areas with better habitat quality and availability [28–30]. In fact, raptors and mammalian communities vary in relation to habitat transformations in several biomes (e.g. forests, deserts, savannah) [28–32]. For example, in Cerrado, the typical savannah in central Brazil, studies have shown that populations of raptors, including scavengers, are more abundant and have more species in areas dominated by natural habitat [29, 32]. Hence, communities of scavengers are expected to be more diverse and rich in road sections surrounded by natural and semi-natural habitats [28–31, 33–35].

Carcass detectability, i.e. the probability of a carcass being detected given it persists to the time of surveys, is highly dependent on the survey method (e.g. driving or walking), observer experience and the body size of carcass [18, 19, 36]. Surveys performed by car generally detect a lower proportion of carcass compared to walking surveys, and this is particularly evident for small-sized species [17, 18, 23]. Yet, disparate detectability values even for the same taxa, have been reported. For example, the detectability of bird carcasses can range between 1 and 67% (mean 26. 9%) [17, 18, 22, 23, 37].

The main objective of this study was to evaluate the influence of carcass persistence time and detectability when quantifying WVC rates. In particular, we aimed to 1) quantify carcass persistence time and assess how it is influenced by body mass of carcass, road-related characteristics, weather conditions and cover of (semi-)natural habitat (as a proxy of scavenger activity); and 2) estimate carcass detectability when performing road surveys by car. As a final goal, we wanted to (3) estimate the proportion of undetected carcasses after correcting for persistence and detectability bias in our studied roads. We expected the persistence time to be longer for large body-sized species, in roads with low traffic volume, and in periods without rainfall [26]. We also expected higher cover of natural habitat near roads to be related to a lower persistence time. The novelties of this study are the broad spatial scale of the study area and road types surveyed, as well the integration of persistence time and detectability for estimating the ‘true’ mortality rates [19, 26].

## Materials and Methods

No specific permissions were required for our study locations/activities, since it is not necessary field permit to monitoring wildlife-vehicle collision. In addition, the project was executed by the environmental agency of the state, responsible for the environmental monitoring. Lastly, it is not necessary authorization for the collection and transport of animals found dead, to scientific or educational use (Normative Ruling N° 03 of September of 2014—ICMBio, see Article 25). Our study did not involve endangered or protected species.

### Study area

**Fig 1:**
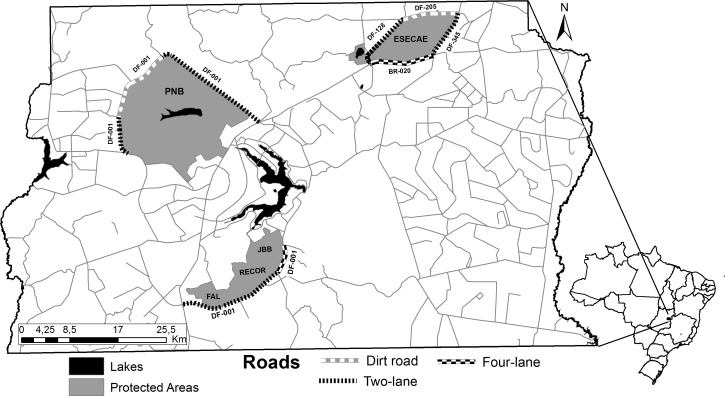
Study area with location of monitored roads and protected areas. Reprinted from Brasilia Environmental Institute (IBRAM) under a CC BY license, with permission from the head of the management of environmental information of IBRAM, original copyright 2016.

This study was conducted in Brasília, within the Federal District, Brazil (Fig 1). The vegetation in the study area is typical of Cerrado biome, and is dominated by savanna forest (“Cerradão” and "Mata de Galeria"), open savanna (“Cerrado *sensu stricto*") and grasslands [38, 39]. The climate is tropical savanna (Köppen-Geiger classification) [40], with an average annual rainfall of 1540mm [41]. The region has distinct dry and wet seasons. During the wet season (October-March), monthly rainfall averages 214mm, monthly temperatures average 21.6°C, and monthly relative air humidity averages 72% [41]. During the dry season (April to September), the monthly rainfall average drops to 41.9mm, monthly temperatures to 19.9°C, and monthly relative air humidity averages 56%, dropping to less than 30% in some periods of the year [41].

The surveys were conducted along nine roads (total 114 km), including four-lane (BR-020 and DF-001, 16 km), two-lane (DF-001, DF-345 and DF-128, 74 km), and dirt roads (DF-205 and DF-001, 24 km) (Fig 1). Both four-lane and two-lane sections were paved (with shoulders). The four-lane roads have the highest traffic volumes (5,000 to 7,000 vehicles/day), the dirt roads have the lowest (33 to 775 vehicles/day), while the two-lane roads have intermediate traffic volumes (775 to 4,000 vehicles/day, with a stretch of 10km reaching 8,000 vehicles/day) [42].These road sections delimit five protected areas, namely Ecological Station of Águas Emendadas-ESECAE (10,000 ha), National Park of Brasília-PNB (44,000 ha), Botanical Garden of Brasilia-JBB (4,000 ha), Experimental Farm of University of Brasília FAL/UnB (4,000 ha), and IBGE Biological Reserve-RECOR (1,300 ha) (Fig 1). UNESCO recognizes all these protected areas as core areas of the Cerrado Biosphere Reserve in the Federal District.

### Data collection

#### Carcass persistence time

Road surveys were performed on a monthly basis, between March 2013 and April 2015, with each survey consisting of five consecutive sampling days (total 26 surveys, 130 sampling days). Three observers (including the driver) in a vehicle at ca. 50km/h sampled repeatedly the five consecutive days searching for carcasses. The vehicle stopped for each carcass found on the road. The observers identified the carcass to the lowest possible taxonomic level, and collected information of the position on the road (lane or shoulder) and the geographic coordinates using a hand-held GPS with 5 m-accuracy. All carcasses were left in the same position in which they had been initially found, and during subsequent sampling days their presence was rechecked to determine persistence time. Hence, carcasses found on the first, second and third days were monitored up to four, three or two days, respectively. Since the surveys were dependent on the technical staff of the local road agency, carcass monitoring could not be performed for more days. However, 5-year data from 484 roadkill surveys in the same roads (5,164 road-killed animals recorded) showed that 60% of carcasses weight less than 100g [43] and, thus, are unlikely to persist on the road for more than three days [17, 19, 26, 44, 45].

#### Carcass detectability

In order to estimate carcass detectability, we randomly selected 500m stretches of the studied roads to be additionally surveyed on foot. These walking surveys were performed independently by another two observers, and began 20 minutes after the car-based team (two observers and one driver in a vehicle at ca. 50km/h) had passed through the selected stretches to avoid visual contact between the car-based and walking teams. Each observer walked along one of the road shoulders looking for carcasses. The same protocol as that of the car-based team for data collection was followed when a carcass was detected. Walking surveys were also performed every month, between May 2013 and April 2015 (total 24 surveys). We surveyed 11 to 12 road stretches in each survey (total 292 stretches, 146 km walked).All carcasses found in the detectability assessment were removed from the road afterwards. The detectability assessment was performed after persistence assessment survey, to avoid removing carcasses that could be recorded in these surveys.

#### Explanatory variables

To assess what factors influence carcass persistence time, we collected additional information on species characteristics, weather conditions and land cover (Table 1). We obtained the mean body mass for each species (S1 Dataset) from bibliographic references [46–52]. Daily rainfall and air humidity were obtained for each survey day from a weather station located at ca. 15 km from the study area, in Brasilia [41]. We used the weather information of the first day a carcass was encountered to characterize the average meteorological conditions during the period of carcass persistence on the road.

**Table 1:** List of explanatory variables and their range values related to the animal, road, weather and land cover used to explain variations in carcass persistence.

Variable	Range
**Animal**	
Body mass (g) ^b^	3–10,000
**Road**	
Position on Road	1: Lane ^a^ 2: Shoulder
Road Type	1: Dirt road (unpaved) ^a^ 2: Two-lane road (paved) 3: Four-lane road (paved)
**Weather**	
**Rainfall**	0: No rain ^a^ 1: Rain event
Air humidity (%) ^c^	0.19–0.92
**Land cover**	
Proportion of savannah ^c^(includes Cerrado *sensu strictu*, open savanna and dense Cerrado)	0.07–0.93
Proportion of forest ^c^ (includes Gallery Forest and "Cerradão")	0.00–0.15
Grasslands and pastures	0.00–0.24
Agriculture	0.00–0.70
**Site**	
Protected area (site) near which was recorded the roadkill ^d^	1—ESECAE 2—PNB 3—JBB/RECOR/FAL

^a^ Reference level in Cox models, see main text.

^b^ Logarithmic transformation.

^c^ Arcsine square root transformation.

^d^ Names of protected areas in study area description.

Land cover information was obtained from a map provided by the Brasília Environmental Institute [53], originated from the multispectral Rapid Eye satellite image from 2011 (spatial resolution of 5m). From this map we extracted the proportion of each land cover type with a circle centered at each carcass location, using buffer sizes of 2, 3 and 4-km radius, which correspond to a total area of ca. 12 to 50 km^2^. We established these buffer sizes in order to capture the variation, in the adjoining areas, of the abundance of the three most common scavengers (obligate or otherwise), namely the southern crested caracara (*Caracara plancus*), the black vulture (*Coragyps atratus*), and the crab-eating fox (*Cerdocyon thous*). These species have estimated home ranges of ca. 7, 15 and 123 km^2^ ^2^, respectively [54, 55, 56].

### Data analyses

We tested for an association between taxonomic Class and body mass using Kruskal-Wallis test. The result obtained revealed a strong relationship (*K* = 110.03, df = 2, p-value < 0.001), with mammals presenting higher body mass than birds and reptiles. Hence, we preferred to work with body mass instead of taxonomic Class, as persistence and detectability of carcasses are more likely similar across similar body sizes than across broad taxonomic levels as Class. To proceed with the analyses, the dataset was divided in carcasses with less than 100g and higher than 100g. This division was based on the dataset of the carcass detectability experiment (see Results and S1 Dataset for detectability experiment dataset). The carcasses that persisted up to the 5^th^ day were classified as right-censored data (i.e., carcasses for which the true persistence time is longer than the study period).

#### Carcass persistence time and influence of environmental variables

The median carcass persistence probability was estimated using the Kaplan-Meier estimator [57], per body mass class and for all records combined. We considered a significant difference if the 95% confidence intervals of median persistence times did not overlap among classes.

Before examining the influence of the explanatory variables (Table 1) on the persistence probability of carcass we checked for pairwise multicollinearity using exploratory plots and Pearson correlations [58]. For each pair of variables exhibiting high correlation (>0.7) [59], the strongest explanatory variable in the simple Cox proportional hazard models was retained for further models (see S1 Table for correlations between variables). We applied, when necessary, arcsine or logarithmic transformations to achieve normality of data [58].

Multivariate mixed Cox models [60] were then fit using all possible combinations of the uncorrelated variables. Model averaging procedures were used to combine results from similarly ranked models (ΔAICc< 2) [61], and to calculate unconditional standard errors for averaged coefficients. Finally, the relative importance of each variable was obtained by summing the Akaike weights for all models (ΔAICc< 2) containing that variable [61]. To evaluate the goodness-of-fit of each model, we used the overall likelihood ratio (LR) test and the proportion of variance explained (R^2^) after visual inspection of model residuals and proportional hazard assumptions.

#### Carcass detectability

To estimate the detectability of carcass surveys performed by car we applied a generalized linear model with binomial error distribution to model the number of detected and non-detected carcasses by the car team, using the function ‘search.efficiency’ available in the R package *carcass* [20]. Body mass was used as explanatory variable. We assumed that the ability to detect carcasses was not remarkably different between observers of both survey teams. This was assessed in joint preliminary surveys, by car and on foot. In all cases, no observer showed a greater capacity or difficulty in detecting carcass on the road.

#### Estimating the ‘real’ number of roadkills

Carcass persistence (s) and detectability (*f*) biases were combined to estimate the detection probability *p* of carcasses following Korner-Nievergelt et al. [62]:

$$p=\frac{f\left(s\frac{1-s_{}^{d}}{1-s}\right)\left({\sum_{i=0}^{n-1}\left(n-i\right)\left(\left(1-f\right)s_{}^{d}\right)}^{i}\right)}{nd}$$ (eq. 1)

Where *n* is the number of searches in the study and *d* is the search interval, i.e. the number of days between consecutive searches. We applied Monte Carlo simulations to account for the uncertainty on the estimation of *p*, using the Korner estimator as implemented in the "Carcass" package [20]. We then estimated the ‘real’ number of carcasses (*N’)* during the survey period, given *p* [20] using the Eq 2, which corresponds to the Horvitz-Thompson estimate [62]:

$$N=\frac{\sum_{i=1}^{n}c_{i}}{p}$$ (eq. 2)

where: *c*_*i*_ is the number of carcass counted during search *i*. N’ was estimated separately for the different body mass classes (i.e., with more or less than 100g).

We did not consider domestic species in the analysis as carcass persistence may have been affected by human action, for example the recovery by owners of road-killed dogs and cats (*pers*. *obs*.).All calculations and plots were performed within the R environment [63]. The R packages *survival* [64] and *coxme* [64] were used in Kaplan-Meier and Cox models, while *carcass* [20] was used in detectability and mortality estimates.

## Results

We collected persistence data for 532 non-domestic road-killed animals, of which 2% were amphibians (n = 14, 2 species), 19% reptiles (n = 101, 31 species), 71% birds (n = 374, 44 species), and 8% mammals (n = 43, 12 species). Three quarters of records (n = 381) were of small size (body mass < 100g) (S1 Dataset). We excluded amphibians from further analyses given the low number of records.

### Carcass persistence time and influence of environmental variables

**Fig 2:**
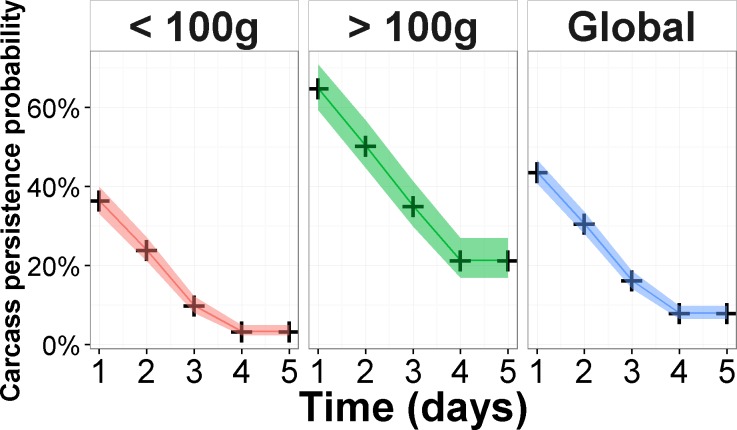
Survival curves from Kaplan-Meier models and corresponding 95% confidence intervals for global data, and body mass classes.

Overall, the median persistence time of carcasses was 2.2 days, with a persistence probability after one day of 0.43 (0.39–0.48, Confidence Interval), dropping to 0.30 (0.27–0.35) in the second day, and reaching a persistence probability of 0.07 (0.05–0.10) in the fourth day. These values indicate a low persistence probability, with a substantial drop beyond the first day (Fig 2 and S2 Table). As expected, the median persistence time was significantly different (no overlapping confidence intervals) between smaller and larger carcasses, being approximately two days for those carcasses with less than 100g and four days for larger ones (S2 Table).

We retained 21 mixed Cox models (ΔAICc<2) relating the persistence time and environmental variables using the information from 3-km buffer radius (Table 2 and Table 3). Each model explained an average of 13.1% (range of12.1–14.5%) of the variance, a low explanatory value. Graphical diagnostics based on the scaled Schoenfeld residuals showed evidence of proportional hazards for all buffers sizes (Figure A, B and C in S1 Appendix). Likewise, the test for proportional hazards was not significant (Table A, B and C in S1 Appendix). Results from models using information for 2 and 4 km buffer radius were similar and are presented in S3 Table and S4 Table, respectively.

**Table 2:** Summary of the top Akaike’s Information Criterion models (ΔAICc<2.0) of the mixed Cox proportional hazard function for persistence data with 3-km buffer radius. All models included site as random effect. LogLik: maximum likelihood value; R^2^: variance explained by the model; ΔAICc: Akaike’s Information Criterion rank; *w*: AIC model weights.

Model	LogLik	R^2^	ΔAICc	*w*
**s+t+b**	-2496.05	0.1285	0	0.09
**s+r+t+b**	-2495.15	0.1317	0.091	0.08
**s+h+t+b**	-2495.37	0.1309	0.622	0.06
**s+g+b**	-2496.88	0.1257	0.890	0.06
**f+s+r+t+b**	-2494.26	0.1347	0.952	0.05
**s+b**	-2497.98	0.1218	0.980	0.05
**f+s+t+b**	-2495.29	0.1312	1	0.05
**f+s+a+r+t+b**	-2493.17	0.1385	1.06	0.05
**f+s+a+t+b**	-2494.24	0.1348	1.18	0.05
**f+s+h+t+b**	-2494.44	0.1341	1.39	0.04
**s+g+r+b**	-2496.15	0.1282	1.48	0.04
**f+s+a+h+t+b**	-2493.34	0.1379	1.49	0.04
**s+a+t+b**	-2495.82	0.1293	1.55	0.04
**s+g+t+b**	-2495.75	0.1296	1.65	0.04
**f+s+g+b**	-2496.17	0.1281	1.65	0.04
**s+a+r+t+b**	-2494.95	0.1324	1.67	0.04
**s+g+r+t+b**	-2494.83	0.1328	1.74	0.04
**s+r+b**	-2497.37	0.124	1.79	0.04
**s+g+h+b**	-2496.32	0.1276	1.83	0.03
**s+r+h+t+b**	-2494.99	0.1322	1.83	0.03
**s+t+p+b**	-2496	0.1287	1.98	0.03

**Legend for models:** a—agriculture; b—body mass; f—forest habitat; g—grasslands; h—air humidity; p—position; r—rainfall; s—savannah; t—road type.

**Table 3:** Model-averaged coefficients (β), respective confidence intervals from unconditional standard errors (95% LCI and 95% UCI), estimates of the hazards ratio (e^β^), and importance value of the top mixed Cox models (ΔAICc<2.0) to 3-km buffer. Variables are ordered according to their importance.

Variable	β	95% LCI	95% UCI	e^β^	Importance
Savannah*	0.803	0.180	1.426	2.26	1.00
Body mass*					1.00
**(>100g)**	-0.192	-0.252	-0.132	0.822	
**Road type**					0.740
**(Two-lane)**	0.007	-0.533	0.551	1.007	
**(Four-lane)**	-0.225	-0.870	0.264	0.795	
**Rainfall**	0.048	-0.065	0.323	1.05	0.370
**Forest habitat**	-0.363	-2.907	0.692	0.690	0.330
**Grasslands**	0.115	-0.362	1.306	1.12	0.240
**Agriculture**	-0.077	-1.002	0.297	0.924	0.220
**Air humidity**	0.068	-0.264	0.890	1.07	0.220
**Position on road**					0.030
**(Shoulder)**	0.001	-0.183	0.224	1.001	

* Significant variables (95% confidence limits).

All 21 models included proportion of savannah habitat and body mass, which were also the variables that attained the highest importance (Tables 2 and 3). According to the averaged model, the persistence time is lower for carcass located in areas with a high cover of savannah habitat nearby and of smaller body mass (<100g) (Table 3). Savannah habitat had the strongest effect on persistence times, with a hazard ratio of 2.26 (Table 3), suggesting a strong effect of the availability of this land use on persistence times. For carcasses with body mass less than 100g, the persistence probability was lower, being 0.36 (0.32–0.41) and 0.03 (0.02–0.05) for the first and fourth days, respectively. For carcasses with larger body mass (>100g), the persistence times were 0.71 (0.65–0.78) and 0.27 (0.22–0.34) for the same time frames (S2 Table).

The remaining variables had no significant coefficient estimates (Table 3). However, the road type was ranked as the third most important variable in model averaging procedures, despite its confidence interval on beta estimate crossing zero (Table 3). Interestingly, most of the top ranked models containing this variable showed a positive effect of the 4-lane road type, when compared to the dirt road. That is, results suggest that persistence time is higher in 4-lane roads relatively to dirt roads.

### Carcass detectability

The walking team detected 117 carcasses, of which 16% were amphibians (n = 19, 2 species), 28% reptiles (n = 33, 12 species), 42% birds (n = 49, 8 species), and 14% mammals (n = 16, 3 species). Of these, only 10 carcasses (6 birds, 2 reptiles and 2 mammals) were also detected by the car-team, corresponding to an overall detectability (*f*) of 10% (6–19% CI). The detectability was apparently lower for carcasses with lower body mass (<100g), 7% (2–15%) relatively to 13.3% (4–29%) for carcasses of larger body mass. However, these results should be considered with caution as their confidence intervals overlapped zero.

### Estimating the ‘real’ number of roadkills

We estimated a *N*’ of 55,906 roadkills/year of small sized species (<100g), which represents a mortality rate of 1.3 roadkills/day/km (Table 4). This estimate was 10 fold higher than the observed value of roadkills. For carcasses of higher body mass, we estimated a *N’* of 5,222 roadkills/year representing 0.12 roadkills/day/km, i.e., a two-fold increase in roadkills numbers. Overall, we estimated a mortality rate of 0.83 roadkills/day/km on our studied roads, representing an annual mortality of 34,536 animals along the 114 km surveyed (Table 4).

**Table 4:** Estimates of total roadkills corrected for biases introduced by carcass persistence and survey method. f–detectability (%), s–estimated median carcass persistence time (days), p–probability of a carcass being detected after one day. N'–mortality estimate with correction for detectability and carcass persistence (roadkills/day/km). C’–mortality estimates without correction for detectability and carcass persistence (roadkills/day/km). Confidence intervals are provided when available.

Group	f	s	p	C’	N'
**Carcass < 100g**	6.8 (2–15)	1.80	0.36 (0.32–0.41)	0.13	1.32 (0.62–3.94)
**Carcass > 100g**	13.3 (4–29)	4.14	0.71 (0.65–0.78)	0.06	0.12 (0.06–0.41)
**Global data**	10 (6–19)	2.15	0.43 (0.39–0.48)	0.15	0.83 (0.47–1.17)

## Discussion

With this study we aimed to evaluate the influence of carcass persistence time and detectability biases in quantifying roadkills. Our results confirm that carcasses persist on roads for about two days, which is in line with previous studies [17, 19, 26, 65]. This is a short persistence period when considering that the periodicity of most roadkill surveys is weekly to monthly. Moreover, our results support that the persistence is largely influenced by environmental variables and characteristics of the road itself, besides the size of the carcass.

The amount of cover of savannah surrounding the roads was the most important predictor explaining the persistence times, hence suggesting a significant effect of scavengers’ activity. We considered that areas with higher savannah coverage have a more diverse and abundant scavenger community and therefore the removal of carcasses by scavengers is likely to be more accentuated in areas of (semi-)natural habitats than in anthropogenic areas (agriculture). This is in agreement with the lower persistence times detected in areas dominated by savannah habitat. Regarding the carcass body size, the persistence time was smaller for small-sized carcasses (<100g), which is in accordance to published literature [19, 26, 66–68]. This lower persistence time of smaller carcasses is likely to be due to a more rapid degradation by passing vehicles [19, 21, 69].The effect of the remaining predictors was generally imprecise as confidence intervals of estimates in model averaging procedures overlapped zero. However, our results suggest a higher persistence for carcasses laying in the four-lane roads when compared to those in dirt roads, which have much less traffic. We suspect that a higher persistence time in 4-lane roads is due to the limited access of scavengers to carrion. That is, higher traffic volume probably inhibit scavengers from attempting to access the carcasses [18, 70]. In fact, a recent study recorded a maximum abundance and diversity of birds of prey along roads with medium traffic volume, when compared to highways with higher traffic volumes [71]. On the other hand, the dirt roads studied are embedded in areas with higher forest cover, hence increasing the chance of carcasses being detected by scavengers. These results stress that the influence of the scavenger-traffic volume relationship on carcass persistence time may not be straightforward [27].Overall, our results highlight that the road mortality rates, as estimated by roadkill surveys, ought to be corrected for scavenger activity, species body mass and road type/traffic volume.

Regarding carcass detectability, our results reveal a low search efficiency of car surveys relatively to walking surveys, particularly for small-sized animals. The detection of smaller animals was two times lower than for larger animals. This difference in detectability between teams is unlikely to be observer-related, as all members received equal training. On the other hand, the car team moved at an average speed of 50km/h, which is probably too fast to detect most small carcasses. Interestingly, the literature reports a wide variability of detectability values, ranging between 1% and 67% [17, 22, 37, 72–74]. Even considering the different taxonomic groups targeted in those studies, the values are still highly discrepant: 4–23% (average 14%) for reptiles [17, 22, 25], 1–67% (27%) for birds [17, 18, 22, 23, 37], and 10–47% (26%) for mammals [17, 18, 22, 75]. Noteworthy, as previously referred the carcass persistence times estimates are similar across those studies, despite the different regions of the world and taxa [17, 21–26, 36, 69]. Hence, we stress the importance of accounting not only for the persistence bias, but perhaps more importantly, for the detectability bias as this latter is more variable across studies. Both are important to be accounted for, the difference is that detectability seems to be more variable and case-specific, so it should be estimated within each study, while persistence might be extrapolated from different areas.

Few studies in road ecology have taken into account carcass persistence and detectability to estimate a more accurate number of ‘real’ mortality rates [17, 18, 22, 23]. As a comparison with our results, a study conducted in the region of Atlantic Forest, in southern Brazil, estimated that corrected estimates for reptile and bird mortalities were 2 to 39 times greater than surveyed values [17]. Our results are in line with these studies and show that in our study region, after correcting for persistence and detectability bias, the actual number of roadkills is likely to be, at least, 2–10 fold greater than estimates based on roadkill surveys. We believe that a more ‘real’ estimate of mortality rates, i.e., corrected by detection and carcass persistence, is the first step to find out if the mortality by roadkills is additive or compensatory [76]. Compensatory mortality hypothesis predicts that no effect on annual survival must occur at low rates of harvest mortality up to a threshold, above which harvest mortality should be additive and with reductions in annual survival [77]. A second step is to identify those species that are likely to experience additive (as opposed to compensatory) mortality from vehicle collisions [76, 78]. The additive population mortality may have worse consequences such as population decreases at short-term [76] what makes conservation strategies priority to the affected species.

It is important to discuss some methodological limitations of our study. First, a low explanatory power of models does not mean that the influence of measured variables is not significant. WVC events are the result of several interrelated factors acting at different scales, from individual behavior responses and experience of both animals and drivers, to the influence of overall landscape connectivity and animal population dynamics. Hence, it is expected that a great proportion of variability is due to stochasticity or to unmeasured variables. Second, our study assumed that all roadkills were detected by walking surveys, but this assumption may not always stand, which could result in an overestimation of detection probabilities [22]. In fact, some road-killed animals are thrown off the lanes at the moment of impact by passing vehicles, and walking observers may fail to notice them [22]. Besides, higher height of the vegetation in shoulders may hide the carcasses and the experience and motivation of the observers may contribute to underestimate in walking surveys [78,79]. However, we are confident that only a small number of carcasses was missed by the walking team, thus having a negligible effect on mortality estimates.

### Management implications

Our study suggests that if surveys are not corrected for carcass persistence and detectability, researchers will significantly underestimate mortality rates. When possible, surveys performed by car should be made at lower speeds. Collinson et al. [79] recommends monitoring by vehicle at speeds at 10–20 km/h. However, lowering the speed survey imply longer survey times, increasing the costs. For the same budget, one would survey less kilometers, which could reduce the generality of the study. These implications perhaps merit further study on ideal sampling design for roadkill surveys to maximize efficiency.

Overall, our results highlight that persistence time is generally concordant across studies, being about two days, although it can vary according to habitat and road type, together with body mass. More importantly, carcass detectability should be estimated for each study, in order to generate less biased mortality rates, as it is apparently the main bias in mortality estimates. We suggest performing an initial training period for observers participating in roadkills surveys to increase observers’ efficiency.

## Supporting Information

S1 Appendix Plots of residuals and results for test of proportional hazard assumptions. (DOCX)

S1 Dataset All Dataset. (XLSX)

S1 Table Results for correlation test for variables with 2, 3 and 4-km buffer radius. (DOCX)

S2 Table Summary of results for persistence estimates. (DOCX)

S3 Table Results for Cox Model to data with 2-km buffer radius. (DOCX)

S4 Table Results for Cox Model to data with 4-km buffer radius. (DOCX)
