# Supplementary material for: Carcass Persistence and Detectability: Reducing the Uncertainty Surrounding Wildlife-Vehicle Collision Surveys
Source: PLoS One. 2016 Nov 2;11(11):e0165608. doi: 10.1371/journal.pone.0165608 (PMC5091900; doi:10.1371/journal.pone.0165608)
Supplement: S1 Appendix — (DOCX) [file pone.0165608.s001.docx]

**S1 Appendix:** Plots of residuals and results for test of proportional hazard assumptions.

**
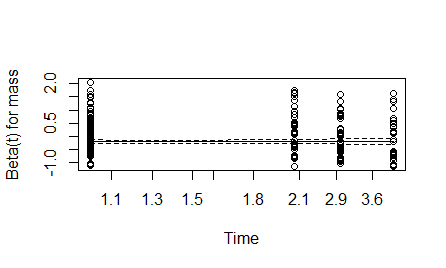
A
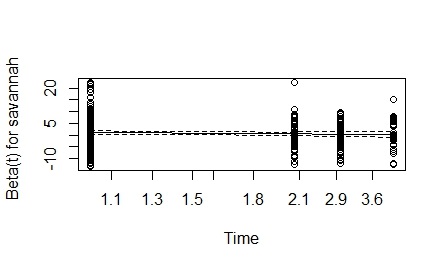
B**

**
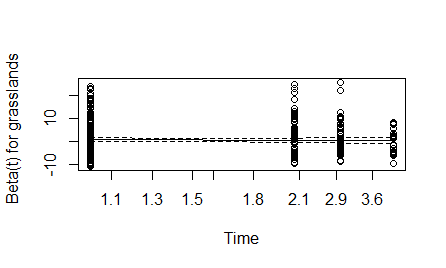
C**

**Figure S4 A.** Plots of scaled Schoenfeld residuals against transformed time for each covariate to the best model with 2-km buffer-size. The solid line is a smoothing spline fit to the plot, with the broken lines representing a ± 2-standard-error band around the fit.

**Table S4 A.** Results for test of the proportional-hazards assumption to the best model with 2-km buffer-size. Chisq: Chi-square test.

|  | **rho** | **Chisq** | **p-value** |
| --- | --- | --- | --- |
| **Body mass** | 0.0136 | 0.0819 | 0.775 |
| **Savannah** | -0.0564 | 1.4884 | 0.222 |
| **Grasslands** | -0.0184 | 0.1666 | 0.683 |
| **GLOBAL** | NA | 1.539 | 0.673 |

**
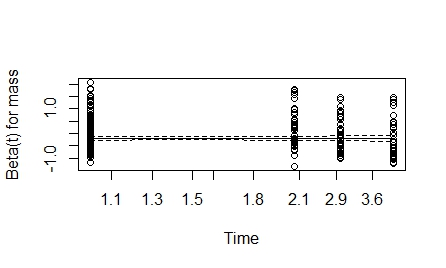
A
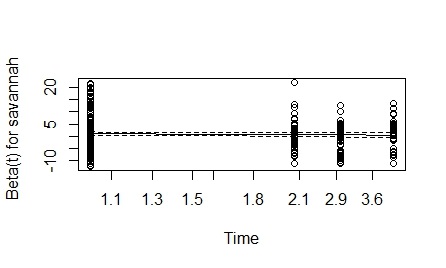
B**

**
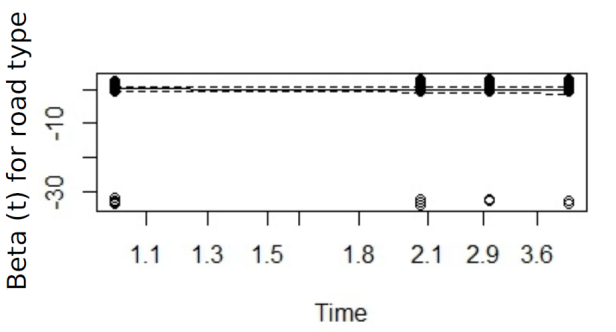
C**

**Figure S4 B.** Plots of scaled Schoenfeld residuals against transformed time for each covariate to the best model with 3-km buffer-size. The solid line is a smoothing spline fit to the plot, with the broken lines representing a ± 2-standard-error band around the fit.

**Table S4 B.** Results for test of the proportional-hazards assumption to the best model with 3-km buffer-size. Chisq: Chi-square test.

|  | **rho** | **Chisq** | **p-value** |
| --- | --- | --- | --- |
| **Body mass** | 0.003 | 0.005 | 0.945 |
| **Savannah** | -0.041 | 0.715 | 0.398 |
| **Two-lane** | -0.028 | 0.371 | 0.543 |
| **Four-lane** | -0.010 | 0.042 | 0.837 |
| **GLOBAL** | NA | 1.685 | 0.793 |

**
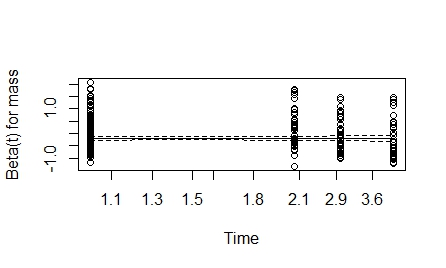
A
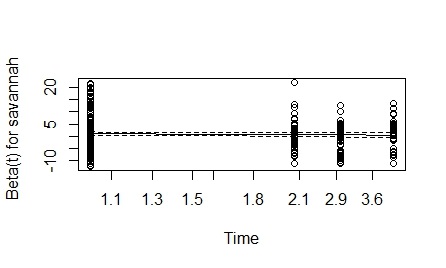
B**

**
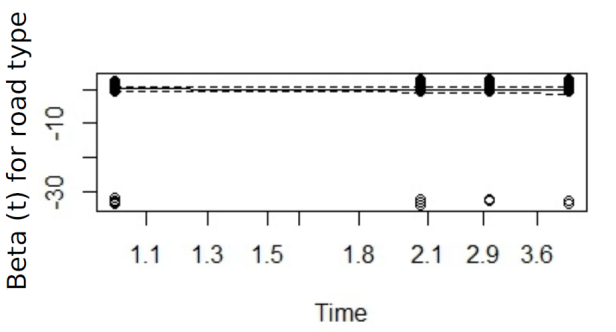
C**

**Figure S4 C.** Plots of scaled Schoenfeld residuals against transformed time for each covariate to the best model with 4-km buffer-size. The solid line is a smoothing spline fit to the plot, with the broken lines representing a ± 2-standard-error band around the fit.

**Table S4 C.** Results for test of the proportional-hazards assumption to the best model with 4-km buffer-size. Chisq: Chi-square test.

|  | **rho** | **Chisq** | **p-value** |
| --- | --- | --- | --- |
| **Body mass** | 0.00471 | 0.00961 | 0.922 |
| **Savannah** | -0.04389 | 0.80955 | 0.368 |
| **Two-lane** | -0.02975 | 0.40977 | 0.522 |
| **Four-Lane** | -0.01038 | 0.05009 | 0.823 |
| **GLOBAL** | NA | 1.83229 | 0.767 |
