# Supplementary material for: Carcass Persistence and Detectability: Reducing the Uncertainty Surrounding Wildlife-Vehicle Collision Surveys
Source: PLoS One. 2016 Nov 2;11(11):e0165608. doi: 10.1371/journal.pone.0165608 (PMC5091900; doi:10.1371/journal.pone.0165608)
Supplement: S1 Table — (DOCX) [file pone.0165608.s003.docx]

**S1Table:** Results for correlation test for variables with 2, 3 and 4-km buffer radius

**S1 Table A.** Results for correlation test for variables with 2-km buffer radius.

| **Variable** | **Rainfall** | **Air humidity** | **Savannah** | **Forest** | **Agriculture** | **Grasslands** | **Body mass** |
| --- | --- | --- | --- | --- | --- | --- | --- |
| **Rainfall** | 1.000 | 0.253 | 0.027 | -0.004 | -0.038 | 0.005 | -0.008 |
| **Air humidity** | 0.253 | 1.000 | 0.066 | -0.012 | -0.003 | -0.090 | -0.060 |
| **Savannah** | 0.027 | 0.066 | 1.000 | -0.224 | -0.327 | -0.224 | 0.043 |
| **Forest** | -0.004 | -0.012 | -0.224 | 1.000 | -0.254 | -0.134 | -0.012 |
| **Agriculture** | -0.038 | -0.003 | -0.327 | -0.254 | 1.000 | -0.114 | -0.033 |
| **Grasslands** | 0.005 | -0.090 | -0.224 | -0.134 | -0.114 | 1.000 | 0.028 |
| **Body mass** | -0.008 | -0.060 | 0.043 | -0.012 | -0.033 | 0.028 | 1.000 |

**S1 Table B.** Results for correlation test for variables with 3-km buffer radius.

| **Variable** | **Rainfall** | **Air humidity** | **Savannah** | **Forest** | **Agriculture** | **Grasslands** | **Body mass** |
| --- | --- | --- | --- | --- | --- | --- | --- |
| **Rainfall** | 1.000 | 0.253 | 0.017 | 0.019 | -0.007 | -0.032 | -0.008 |
| **Air humidity** | 0.253 | 1.000 | 0.069 | 0.057 | -0.026 | -0.075 | -0.060 |
| **Savannah** | 0.017 | 0.069 | 1.000 | -0.178 | -0.221 | -0.336 | 0.056 |
| **Forest** | 0.019 | 0.057 | -0.178 | 1.000 | -0.394 | -0.042 | 0.006 |
| **Agriculture** | -0.007 | -0.026 | -0.221 | -0.394 | 1.000 | -0.080 | -0.029 |
| **Grasslands** | -0.032 | -0.075 | -0.336 | -0.042 | -0.080 | 1.000 | -0.006 |
| **Body mass** | -0.008 | -0.060 | 0.056 | 0.006 | -0.029 | -0.006 | 1.000 |

**S1 Table C.** Results for correlation test for variables with 4-km buffer radius.

| **Variable** | **Rainfall** | **Air humidity** | **Savannah** | **Forest** | **Agriculture** | **Grasslands** | **Body mass** |
| --- | --- | --- | --- | --- | --- | --- | --- |
| **Rainfall** | 1.000 | 0.253 | 0.009 | 0.004 | 0.005 | -0.058 | -0.008 |
| **Air humidity** | 0.253 | 1.000 | 0.057 | 0.029 | -0.021 | -0.040 | -0.060 |
| **Savannah** | 0.009 | 0.057 | 1.000 | 0.015 | -0.136 | -0.516 | 0.055 |
| **Forest** | 0.004 | 0.029 | 0.015 | 1.000 | -0.471 | 0.042 | -0.003 |
| **Agriculture** | 0.005 | -0.021 | -0.136 | -0.471 | 1.000 | -0.207 | -0.010 |
| **Grasslands** | -0.058 | -0.040 | -0.516 | 0.042 | -0.207 | 1.000 | -0.013 |
| **Body mass** | -0.008 | -0.060 | 0.055 | -0.003 | -0.010 | -0.013 | 1.000 |
