# Supplementary material for: Carcass Persistence and Detectability: Reducing the Uncertainty Surrounding Wildlife-Vehicle Collision Surveys
Source: PLoS One. 2016 Nov 2;11(11):e0165608. doi: 10.1371/journal.pone.0165608 (PMC5091900; doi:10.1371/journal.pone.0165608)
Supplement: S2 Table — (DOCX) [file pone.0165608.s004.docx]

**S2 Table:** Summary of results for persistence estimates

**S2 Table.** Summary of results for persistence estimates for each body mass class and the ‘‘global data’’. N: sample size; Mean (95% CI): mean persistence time probabilities; T=1, T=2, T=3, T=4: estimate of persistence probability for 1-day (T=1), 2-day (T=2), 3-day (T=3) and 4-day (T=4) and corresponding 95% confidence intervals obtained with a Kaplan-Meier estimator.

| **Groups** | **N** | **Mean Persistence Time (days)** | **T=1** | **T=2** | **T=3** | **T=4** |
| --- | --- | --- | --- | --- | --- | --- |
| **WVC < 100g*** | 316 | 1.80 | 0.36 (0.32-0.41) | 0.24 (0.20-0.29) | 0.09(0.07-0.13) | 0.03(0.02-0.05) |
| **WVC >100g**** | 199 | 4.14 | 0.71 (0.65-0.78) | 0.57 (0.51-0.64) | 0.42 (0.36-0.50) | 0.27 (0.22-0.34) |
| **Global data** | 515 | 2.15 | 0.43 (0.39-0.48) | 0.30 (0.27-0.35) | 0.16 (0.13-0.19) | 0.07 (0.05-0.10) |

* Carcass with body mass less than 100g

** Carcass with body mass higher than 100g
