# Supplementary material for: Carcass Persistence and Detectability: Reducing the Uncertainty Surrounding Wildlife-Vehicle Collision Surveys
Source: PLoS One. 2016 Nov 2;11(11):e0165608. doi: 10.1371/journal.pone.0165608 (PMC5091900; doi:10.1371/journal.pone.0165608)
Supplement: S3 Table — (DOCX) [file pone.0165608.s005.docx]

**S3 Table:** Results for Cox Model to data with 2-km buffer radius.

**S3 Table A.**  Summary of the top Akaike’s Information Criterion models (ΔAICc<2.0) of the Cox proportional hazard function for persistence data with 2-km buffer radius. LL test: maximum likelihood test; R^2^: variance explained by the model; ΔAICc: Akaike’s Information Criterion rank; *w*: AIC model weights.

| **Model** | **LogLik** | **R ^2^** | **ΔAICc** | ***w*** |
| --- | --- | --- | --- | --- |
| **s+g+b** | -2495.53 | 0.1304 | 0 | 0.12 |
| **s+g+r+b** | -2494.64 | 0.1334 | 0.27 | 0.11 |
| **s+g+h+b** | -2494.76 | 0.133 | 0.53 | 0.09 |
| **f+s+g+b** | -2494.91 | 0.1325 | 0.91 | 0.08 |
| **f+s+g+r+b** | -2493.93 | 0.1359 | 0.99 | 0.07 |
| **s+g+r+t+b** | -2493.47 | 0.1374 | 1.17 | 0.07 |
| **f+s+g+h+b** | -2494.1 | 0.1353 | 1.35 | 0.06 |
| **s+g+t+b** | -2494.58 | 0.1336 | 1.45 | 0.06 |
| **f+s+a+r+t+b** | -2492.61 | 0.1404 | 1.68 | 0.05 |
| **s+g+h+t+b** | -2493.69 | 0.1367 | 1.69 | 0.05 |
| **f+s+g+r+t+b** | -2492.58 | 0.1405 | 1.71 | 0.05 |
| **s+a+g+b** | -2495.44 | 0.1307 | 1.87 | 0.05 |
| **s+g+r+h+b** | -2494.44 | 0.1341 | 1.93 | 0.05 |
| **s+r+t+b** | -2495.12 | 0.1318 | 1.94 | 0.05 |
| **f+s+a+g+b** | -2494.4 | 0.1342 | 1.98 | 0.05 |

**Legend for models:** a - agriculture; b - body mass; f - forest habitat; g - grasslands; h - air humidity; p - position; r - rainfall; s - savannah; t - road type.

**S3 Table B.** Model-averaged coefficients (β), respective confidence intervals from unconditional standard errors (95% LCI and 95% UCI), estimates of the hazards ratio (e^β^), and importance value (Importance) of the top mixed Cox models (ΔAICc<2.0) to 2-km buffer radius. Variables are ordered according to Importance.

| **Variable** | **β** | **95% LCI** | **95% UCI** | **e^β^** | **Importance** |
| --- | --- | --- | --- | --- | --- |
| **Savannah*** | 0.874 | 0.207 | 1.540 | 2.43 | 1.00 |
| **Body mass*** | -0.194 | -0.254 | -0.134 | 0.820 | 1.00 |
| **Grassalands** | 0.692 | 0.030 | 1.506 | 2.02 | 0.90 |
| **Rainfall** | 0.061 | -0.059 | 0.332 | 1.06 | 0.44 |
| **Forest habitat** | -0.293 | -2.172 | 0.554 | 0.741 | 0.36 |
| **Road type** |  |  |  |  | 0.33 |
| **(Two-lane)** | -0.005 | -0.556 | 0.528 | 0.994 |  |
| **(Four-lane)** | -0.093 | -0.860 | 0.292 | 0.909 |  |
| **Air humidity** | 0.082 | -0.252 | 0.899 | 1.08 | 0.25 |
| **Agriculture** | -0.039 | -0.853 | 0.316 | 0.961 | 0.15 |
| **Position on road** |  |  |  |  |  |
| **(Shoulder)** | 0.00 | 0.00 | 0.00 | 0.00 | 0.00 |

***** Significant variables (95% confidence limits)
