# Supplementary material for: Carcass Persistence and Detectability: Reducing the Uncertainty Surrounding Wildlife-Vehicle Collision Surveys
Source: PLoS One. 2016 Nov 2;11(11):e0165608. doi: 10.1371/journal.pone.0165608 (PMC5091900; doi:10.1371/journal.pone.0165608)
Supplement: S4 Table — (DOCX) [file pone.0165608.s006.docx]

**S4 Table:** Results for Cox Model to data with 4-km buffer radius.

**S4 Table A.**  Summary of the top Akaike’s Information Criterion models (ΔAICc<2.0) of the Cox proportional hazard function for persistence data with 4-km byffer radius. LL test: maximum likelihood test; R^2^: variance explained by the model; AICc: Akaike’s Information Criterion; ΔAICc: Akaike’s Information Criterion rank; *w*: AIC model weights.

| **Model** | **LogLik** | **R ^2^** | **AICc** | **ΔAICc** | ***w*** |
| --- | --- | --- | --- | --- | --- |
| s+t+b | -2496.41 | 0.1273 | 5002.89 | 0 | 0.1 |
| s+r+t+b | -2495.47 | 0.1305 | 5002.92 | 0.03 | 0.1 |
| s+h+t+b | -2495.67 | 0.1299 | 5003.4 | 0.5 | 0.08 |
| s+b | -2498.23 | 0.121 | 5003.56 | 0.67 | 0.07 |
| s+g+b | -2497.26 | 0.1243 | 5003.79 | 0.9 | 0.06 |
| f+s+r+t+b | -2494.74 | 0.1331 | 5003.96 | 1.07 | 0.06 |
| f+s+t+b | -2495.76 | 0.1295 | 5004.01 | 1.12 | 0.06 |
| s+r+b | -2497.58 | 0.1232 | 5004.29 | 1.4 | 0.05 |
| s+g+r+b | -2496.52 | 0.1269 | 5004.37 | 1.47 | 0.05 |
| f+s+h+t+b | -2494.96 | 0.1323 | 5004.45 | 1.56 | 0.04 |
| s+h+b | -2497.68 | 0.1229 | 5004.5 | 1.61 | 0.04 |
| f+s+g+b | -2496.58 | 0.1267 | 5004.57 | 1.68 | 0.04 |
| s+r+h+t+b | -2495.29 | 0.1312 | 5004.62 | 1.72 | 0.04 |
| s+g+h+b | -2496.7 | 0.1263 | 5004.71 | 1.82 | 0.04 |
| s+g+t+b | -2496.27 | 0.1278 | 5004.73 | 1.84 | 0.04 |
| s+a+t+b | -2496.35 | 0.1275 | 5004.76 | 1.87 | 0.04 |
| s+g+r+t+b | -2495.34 | 0.131 | 5004.78 | 1.89 | 0.04 |
| s+a+r+t+b | -2495.43 | 0.1307 | 5004.83 | 1.94 | 0.04 |
| s+t+p+b | -2496.35 | 0.1275 | 5004.87 | 1.98 | 0.04 |

**Legend for models:** a - agriculture; b - body mass; f - forest habitat; g - grasslands; h - air humidity; p - position; r - rainfall; s - savannah; t - road type.

**S4 Table B.** Model-averaged coefficients (β), respective confidence intervals from unconditional standard errors (95% LCI and 95% UCI), estimates of the hazards ratio (e^β^), and importance value (Importance) of the top mixed Cox models (ΔAICc<2.0) to 4-km buffer size. Variables are ordered according to Importance.

| **Variable** | **Level** | **β** | **95% LCI** | **95% UCI** | **e^β^** | **Importance** |
| --- | --- | --- | --- | --- | --- | --- |
| **Savannah*** |  | 0.859 | 0.175 | 1.542 | 2.39 | 1.00 |
| **Body mass*** |  | -0.190 | -0.250 | -0.130 | 0.824 | 1.00 |
| **Road type** |  |  |  |  |  | 0.65 |
| **(Two-lane)** |  | 0.021 | -0.510 | 0.575 | 1.02 |  |
| **(Four-lane)** |  | -0.178 | -0.837 | 0.290 | 0.426 |  |
| **Rainfall** |  | 0.046 | -0.067 | 0.321 | 1.04 | 0.36 |
| **Grassalands** |  | 0.112 | -0.483 | 1.327 | 1.12 | 0.26 |
| **Air humidity** |  | 0.073 | -0.271 | 0.877 | 1.07 | 0.24 |
| **Forest habitat** |  | -0.173 | -2.725 | 0.976 | 0.838 | 0.20 |
| **Agriculture** |  | -0.010 | -0.809 | 0.552 | 0.989 | 0.07 |
| **Position** |  |  |  |  |  |  |
| **(shoulder)** |  | 0.001 | -0.183 | 0.224 | 1.001 | 0.04 |

***** Significant variables (95% confidence limits)
